# Metabolic Problems Among People With Treatment-Resistant Schizophrenia are Not Unique to Clozapine

**DOI:** 10.1097/JCP.0000000000002058

**Published:** 2025-08-15

**Authors:** Alexander Panickacheril John, Hitesh Prajapati, Milan Dragovic

**Affiliations:** 1 The University of Western Australia & Bentley Health Service; 2 Bentley Health Service; 3 North Metropolitan Health Service, Graylands Hospital, Perth, Australia

**Keywords:** clozapine, metabolic syndrome, antipsychotics

## Abstract

**Background:**

It is unclear whether treatment with clozapine, when compared directly with other antipsychotics, is differentially associated with metabolic problems among people with treatment-resistant schizophrenia (TRS). We evaluated the prevalence of diabetes, dyslipidemia, obesity, and hypertension, and parameters such as glucose, lipids, weight, and blood pressure of people with TRS managed on clozapine and non-clozapine antipsychotics.

**Methods:**

Demographic, clinical, and metabolic data of patients with TRS who commenced clozapine between 2006 and 2016 at a public hospital were collected in 2023. Participants were divided into those who were on clozapine and those who had discontinued clozapine and were on other antipsychotics when the data were collected. Metabolic disorders of the 2 groups were compared with the *t* test, the χ^2^ test, and the Mann-Whitney *U* test. The significance level was set at *P*<0.05.

**Results:**

One hundred six participants with TRS were on treatment with clozapine, and 41 with other antipsychotics. The participants’ mean age was 44.3 (SD: 10.2) years, and 74.1% were males. Metabolic disorders such as dyslipidemia (45.6%), obesity (37.4%), and diabetes (22.1%) were prevalent among people with TRS. However, there was no significant difference in diabetes, obesity, and hypertension, and parameters such as blood sugar, lipid levels, and blood pressure between the clozapine and non-clozapine cohorts. While dyslipidaemia was significantly higher among those who were managed on clozapine, 75% of them were prescribed statins.

**Conclusions:**

A comprehensive approach addressing a broad range of risk factors, rather than solely focusing on clozapine, could be beneficial when evaluating and treating metabolic problems among people with TRS.

Robust evidence gathered through multiple research studies suggests that people with schizophrenia have significantly higher odds of developing a range of metabolic problems such as obesity, diabetes, dyslipidemia, hypertension, and metabolic syndrome.^1,2^ These metabolic disorders account for a significant proportion of the excess deaths due to natural causes, particularly due to higher rates of death due to cardiovascular and respiratory diseases among people with schizophrenia.^3^ The origins of increased metabolic problems among people with schizophrenia are multifactorial.^4^ Several environmental and lifestyle risk factors for metabolic syndrome, diabetes, obesity, and dyslipidemia, such as significantly increased time spent in sedentary behavior, participating less in moderate and vigorous physical exercise, unhealthy eating habits, consuming a diet rich in high-energy food and poor in fiber and fruit content, and high rates of smoking and substance abuse, are common in people with schizophrenia.^4–7^ Furthermore, it has been proposed that there are several shared complex genetic factors, neuroendocrine characteristics, and inflammatory mechanisms between metabolic syndrome, diabetes, and schizophrenia.^8,9^ In addition, in the past few decades, there has been significant interest among researchers and clinicians in scrutinizing the role of antipsychotics, in particular second-generation antipsychotics, in the genesis of metabolic problems such as obesity, diabetes, dyslipidemia, and metabolic syndrome.^1,2^ Different antipsychotics have different affinities on dopamine, serotonin, muscarinic, adrenergic, and histamine receptors and other molecular targets, which are believed to be responsible for their diverse metabolic adverse effects through their actions on target organs.^10,11^

Several systematic reviews of randomized controlled trials and population-based longitudinal studies have concluded that among antipsychotics, clozapine ranks among those with the worst metabolic profile, and is more likely to be associated with metabolic issues such as obesity, diabetes, dyslipidemia, and metabolic syndrome than other antipsychotics.^1,2,11–15^ For instance, Mitchell et al,^1^ in their meta-analysis, concluded that the rate of metabolic syndrome was 51.9% for clozapine, 28.2% for olanzapine, 27.9% for risperidone, and 20.2% for unmedicated patients with schizophrenia. A study from a clozapine clinic in Australia found that 58.3% of patients treated with clozapine met the criteria for metabolic syndrome. In all, 79.6% were overweight or obese, 55.9% had blood pressure meeting metabolic syndrome criteria, 46.6% had elevated fasting blood glucose, and 55.2% had elevated triglycerides.^16^ Some authors have suggested that clozapine’s wide-ranging receptor affinity and its effect on inflammatory markers and signaling pathways, alteration of ghrelin and leptin release, oxidative stress reaction, and direct effect on insulin receptors of pancreatic beta cells increase the risk of metabolic problems.^17,18^ However, teasing apart the individual contributions of poor diet, lack of exercise, unhealthy habits such as smoking and substance abuse, and direct effects of the antipsychotic is quite challenging.^4,18^

A significant proportion of people with schizophrenia, deemed to be around 25% in first-episode and 40% in multi-episode schizophrenia, are considered to have treatment-resistant schizophrenia (TRS).^19,20^ Clozapine is the gold standard for treating people with TRS and is the medication that has been universally recommended for managing this condition by various treatment guidelines for schizophrenia.^21^ While other antipsychotics are used for a broad range of people with schizophrenia and other psychotic disorders, apprehension about potentially lethal adverse effects, the common occurrence of multiple side effects, concern about metabolic adverse effects, and difficulties in adhering to the mandatory monitoring regimes have resulted in the clinical use of clozapine being restricted predominantly to people with TRS in many countries.^21–23^ TRS is associated with more severe psychosocial dysfunctions, higher rates of substance abuse, severity of symptoms, worse community functioning, and the presence of inflammatory markers compared with treatment-responsive schizophrenia.^24–26^ It is possible that some of the adverse psychosocial, lifestyle, clinical, and inflammatory factors associated with TRS could enhance the risk of metabolic abnormalities among people with TRS compared with those with treatment-responsive schizophrenia. Hence, it is possible that people with TRS could experience higher rates of metabolic problems irrespective of the nature of the antipsychotics being used for the treatment of their condition. However, studies have rarely examined the prevalence of metabolic disorders and metabolic parameters among people with TRS treated with clozapine in comparison to those treated with other antipsychotics. We examined the prevalence of obesity, diabetes, dyslipidemia, fasting blood sugar and lipid levels, and weight and blood pressure measurements among patients with TRS who were treated with clozapine or other antipsychotics.

## METHODS

### Study Location

This study was conducted at Bentley Adult Community Mental Health Service, which is located in the southeast metropolitan area of Perth, Western Australia. This public mental health service catered to a population of ~300,000 adults living within its geographical catchment area and, at the time of the study, had around 650 active patients with severe mental illness registered with its community mental health clinic. In addition to other clinical services, the community mental health service ran a Clozapine Clinic with 2 full-time mental health nurses and several psychiatrists, providing clinical care to all adult patients treated with clozapine. For the patients managed on clozapine, the hospital guideline was to review patients weekly for the first 18 weeks of its commencement and, after 18 weeks, every 4 weeks at the Clozapine Clinic. Detailed clinical notes were entered at each visit by the clozapine nurses and psychiatrists. At each visit, blood pressure, heart rate, and weight were monitored, and the monitoring guideline recommended that fasting blood glucose and lipids be done every 6 months at accredited pathology laboratories.

### Participants’ Inclusion and Exclusion Criteria

The study sample comprised patients who were managed through the Clozapine Clinic of Bentley Adult Community Mental Health Service with a diagnosis of schizophrenia or schizoaffective disorder, who were commenced on clozapine between January 1, 2006, and December 31, 2016, and were active with the service at the time of collection of the data. The indication for commencement of clozapine in Australia is for treatment-resistant schizophrenia (www.tga.org.au). If the patients were commenced on clozapine for another diagnosis, they were excluded from the study. Data for this study were collected from February 1, 2023, to April 30, 2023. Both patients who continued clozapine and discontinued clozapine after its commencement were included in the study. However, patients who had discontinued clozapine and had <6 months of exposure to other antipsychotics at the time of the data collection were not included in this research.

### Data Collection and Measures

Medical records, electronic records, and laboratory investigation findings of the included patients were accessed, and relevant data were collected by a senior psychiatry registrar in the final year of his 5-year psychiatry postgraduate training. A senior psychiatrist provided supervision during the data collection process. The data collected included demographic details such as age, sex, and occupation, clinical data such as The Diagnostic and Statistical Manual of Mental Disorders, 5th edition^27^ diagnosis, presence of medical and psychiatric comorbidities, current substance use (past 3 months), number of hospitalizations in the lifetime and past 2 years, number of incidents of aggression toward others or self-harm in the past 6 months, and duration from the onset of psychotic symptoms up to the period of collection of data. We also collected information on the current clozapine dosage, most recent clozapine levels, time since cessation of clozapine (among those who discontinued clozapine), and details of other antipsychotics prescribed. In addition, we collected data on the latest metabolic parameters available, including weight, body mass index (BMI), blood pressure (BP, systolic/diastolic), and fasting blood glucose and lipid levels.

Prescriptions of medications for general medical conditions such as diabetes, hypertension, dyslipidemia, and weight management were collected from the medical records. Clear documentation of a diagnosis of diabetes, dyslipidemia, or hypertension in the medical records of the patients by psychiatrists, general practitioners, or specialists, and supporting evidence through the results of investigations or prescriptions of medications for hypertension, dyslipidemia, and diabetes were considered as indicative of the presence of the relevant medical comorbidity. However, treatment with agents such as metformin, if it was explicitly for weight management, was not considered indicative of diabetes. Body mass index (kg/m^2^) was classified as healthy weight (18.5 to 24.9), overweight (25 to 29.9), and obesity (30.0 and above) (www.cdc.gov/bmi/adult-calculator).

### Ethics Approval

This study was part of a research project titled “Prevalence of antipsychotic polypharmacy among patients with treatment-resistant schizophrenia managed on clozapine or other antipsychotics,” which received ethics and governance approval from the Human Research Ethics and Governance Committees of Royal Perth Hospital, Western Australia (Project Reference Number RGS0000004367). The ethics committee provided a waiver from obtaining consent from individual participants for accessing medical records, collecting the selected data, and publishing non-identifiable data.

### Data Analysis

We divided the TRS patients into 2 groups: those patients who were on clozapine at the time of the data collection (clozapine group) and those who had discontinued clozapine and were being treated with other antipsychotics for at least 6 months (non-clozapine group). Data were analyzed using the statistical software Jamovi (2.2.5). Descriptive statistics summarized continuous variables using means and SDs, or, in the case of variables that did not conform to a normal distribution, as medians with interquartile ranges. Proportions were used to describe categorical variables. Study groups were compared with the independent samples *t* test and the χ^2^ test. When the assumption of normality was violated, between-group differences were examined using nonparametric alternatives—that is, the Mann-Whitney *U* test. We used the false-discovery rate method to adjust *p* values given the necessity for multiple comparisons within the same data set.^28^ A 2-tailed significance of *P*<0.05 was used.

## RESULTS

### Composition of the Study Cohorts

One hundred forty-seven participants met the criteria for inclusion in the study. The mean age of the participants was 44.3 (SD: 10.2) years, and 74.1% were males. Nearly 90% were unemployed and supported by the government through disability pension, indicative of the severity of functional impairments associated with our cohort of TRS patients. One hundred thirty-one (89.1%) participants were diagnosed with schizophrenia and 16 (10.9%) with schizoaffective disorder. Nearly 70% had comorbid DSM-5 psychiatric diagnoses, with substance abuse (41%), anxiety disorders (26%), and depressive disorders (16%) being the most frequent. Medical comorbidities were also common, with only 15.6% not diagnosed with a comorbid medical condition. Metabolic problems such as dyslipidemia (45.6%), obesity (37.4%), diabetes (22.1%), and hypertension (16.3%) were common among the cohort of TRS patients. The participants had a chronic illness with a mean duration of psychosis of 19.4 (SD: 8.8) years, and patients who were on clozapine were on treatment with this medication for a mean duration of 10.6 (SD: 9.0) years. The sample had many lifetime hospitalizations (mean: 10.3, SD: 8.6), indicative of the severity of the illness. The mean clozapine dose at the time of data collection was 352 mg (SD: 152), the mean plasma clozapine level was 345 µg/L (SD: 314), and the norclozapine level was 170 µg/L (SD: 166) among those who were treated with clozapine.

Out of the 147 participants with TRS, 106 were treated with clozapine, and 41 were managed with other antipsychotics. Those participants in the non-clozapine group had ceased clozapine for an average duration of 5.5 (SD: 3.8, range: 0.6 to 15.6) years before the commencement of the study. Those in the non-clozapine group were managed on other second-generation antipsychotics such as aripiprazole (31.7%), olanzapine (68.3%), paliperidone (34.1%), quetiapine (26.8%), and risperidone (7.3%). Twenty-two percent were on treatment with the first-generation antipsychotic, zuclopenthixol decanoate.

### Comparison of the Demographic, Clinical, and Treatment Characteristics of the Clozapine and Non-Clozapine Groups of TRS Participants

While sociodemographic characteristics such as age, sex, and proportion of participants who were unemployed and on disability support pension were similar between the groups, certain types of substance abuse, particularly use of stimulants and cannabis, were significantly more prevalent among people with TRS who were not managed on clozapine (Table 1). Furthermore, clinical outcomes such as the number of hospitalizations in the past 2 years and the incidence of aggression and self-harm in the past 6 months were lower among those who were on treatment with clozapine. Those participants with TRS who were in the non-clozapine group were being treated with a greater number of antipsychotics (Table 2). While 33 (80.5%) participants were on antipsychotic polypharmacy (concurrent use of 2 or more regular antipsychotics) in the non-clozapine group, the rates were significantly lower in the clozapine group at 55.7% (χ^2^=7.78, *P*=0.005). However, the rate of use of other psychotropics, such as antidepressants, mood stabilizers, and benzodiazepines, was similar between the 2 groups. While the numbers receiving prescriptions for treatment of diabetes and hypertension were similar between the groups, the proportion of people who were prescribed statins was double in the clozapine group compared with the participants not treated with clozapine. The difference between the 2 groups in statin use just failed to reach significance at *P*=0.054.

**TABLE 1:** Demographic and Clinical Characteristics of People With Treatment-Resistant Schizophrenia Who Were on Treatment With Clozapine or Other Antipsychotics (n=147)

Variables	On Clozapine, n=106	Not on Clozapine, n=41	Test	*P*
Sex, n (%)				
Male	81 (76.4)	28 (68.3)	1.02*	0.268
Female	25 (23.6)	13 (31.7)		
Age (y), median (±IQR/2)	42.0 (±6.9)	42.0 (±8.5)	1970.5†	0.287
Occupation, n (%)				
Unemployed	90 (84.9)	38 (92.7)		
Employed part-time	8 (7.5)	2 (4.9)	1.99*	0.322
Employed full-time	7 (6.6)	1 (2.4)		
Home duties/student	1 (0.9)	0 (0.0)		
Income source, n (%)				
Own income	14 (13.2)	3 (7.3)		
New start/youth allowance	1 (0.9)	1 (2.4)	1.44*	0.302
DSP	91 (85.8)	37 (90.2)		
Diagnoses, n (%)				
Schizophrenia	92 (86.8)	39 (95.1)	2.12*	0.175
Schizoaffective disorder	14 (13.2)	2 (4.9)		
Duration of psychosis (y), M (SD)	19.1 (8.3)	20.3 (9.9)	0.74‡	0.297
Presence of any psychiatric comorbidity, n (%)	70 (66.0)	33 (80.5)	2.94*	0.111
Presence of any substance abuse, n (%)	38 (35.8)	22 (53.7)	3.88*	0.080
Stimulant abuse, n (%)	8 (7.5)	15 (36.6)	18.89*	**0.000**
Cannabis abuse, n (%)	18 (17.0)	17 (41.5)	9.77*	**0.007**
Alcohol abuse, n (%)	38 (35.8)	19 (46.3)	1.37*	0.242
Smoking	67 (63.2)	29 (70.7)	0.74*	0.281
Hospitalizations, median (±IQR/2)				
Last 2 years	0.0 (±0)	1.0 (±1)	1135.5†	**0.000**
Total number	6.5 (±3)	12.0 (±6)	1192.0†	**0.000**
Aggression incidents, median (±IQR/2)	1.0 (±0.5)	2.0 (±2)	1651.5†	**0.031**
Self-harm incidents, n (%)	6 (5.7)	9 (22.0)	8.56*	**0.012**

Significant *P* values in bold.

* χ^2^ test.

† Mann-Whitney *U* test.

‡ Independent samples *t* test.

**TABLE 2:** Antipsychotic, Other Psychotropics, and Medication Treatment for Metabolic Conditions Among Participants With Treatment-Resistant Schizophrenia Who Were on Treatment With Clozapine or Other Antipsychotics (n=147)

Variables	On Clozapine, n=106	Not on Clozapine, n=41	Test	*P*
Antipsychotics, median (±IQR/2)	2 (±0)	2 (±0.5)	1686.5*	**0.043**
Antidepressants, n (%)	43 (40.6)	10 (24.4)	3.36†	0.093
Mood stabilizers, n (%)	40 (37.7)	11 (26.8)	1.55†	0.240
Benzodiazepines, n (%)	28 (26.4)	15 (36.6)	1.48†	0.237
Antidiabetic, n (%)‡	27 (25.5)	9 (22.0)	0.20†	0.328
Antihypertensive, n (%)	17 (16.0)	8 (19.5)	0.25†	0.326
Statin, n (%)	41 (38.7)	8 (19.5)	4.89†	0.054

Significant *P* values in bold.

* Mann-Whitney *U* test.

† χ^2^ test.

‡ Includes people who were commenced on metformin for weight management.

### Comparison of Metabolic Parameters and Metabolic Disorders Between the Clozapine and Non-Clozapine Groups of TRS Participants

Of note, while metabolic disorders were common among TRS patients, there were no s7ignificant differences in the numbers of patients diagnosed with diabetes, obesity, or hypertension between the groups (Table 3). However, dyslipidemia was significantly higher in the clozapine group, with almost double the rate of those in the non-clozapine group. Results of the measurements of weight, BMI, blood pressure, and fasting blood sugar were similar between the groups. None of the lipid parameters were significantly different between the groups. The mean cholesterol level was higher in the clozapine group by 0.4 mmol/L, and the difference between the groups marginally failed to reach significance (*P*=0.063).

**TABLE 3:** Comparison of Metabolic Parameters and Metabolic Comorbidities Among People With Treatment-Resistant Schizophrenia Who Were on Treatment With Clozapine or Other Antipsychotics (n=147)

Variables	On Clozapine, n=106	Not on Clozapine, n=41	Test	*P*
Weight (kg), M (SD)	97.7 (21.9)	94.2 (16.3)	0.93*	0.277
BMI, median (±IQR/2)	30.6 (±3.6)	29.6 (±4.2)	2047.5†	0.321
Systolic BP (mm Hg), M (SD)	128.2 (14.5)	126.0 (13.0)	0.84*	0.278
Diastolic BP (mm Hg), M (SD)	81.2 (8.6)	80.0 (7.3)	0.80*	0.282
BSL (mmol/L), median (±IQR/2)	5.5 (5.1-5.9)	5.6 (5.3-6.2)	1895.0†	0.236
Cholesterol (mmol/L), M (SD)	4.8 (1.1)	4.4 (0.9)	2.13*	0.063
HDL (mmol/L), median (±IQR/2)	1.1 (±0.2)	1.1 (±0.2)	2066.5†	0.332
Triglyceride (mmol/L), median (±IQR/2)	2.1 (±0.7)	1.9 (±0.6)	1946.5†	0.269
LDL (mmol/L), median (±IQR/2)	2.5 (±0.6)	2.2 (±0.7)	1736.0	0.089
Presence of any comorbidity, n (%)	89 (84.0)	35 (85.4)	0.04*	0.395
Diabetes	21 (19.8)	10 (24.4)	0.37*	0.315
Hypertension	16 (15.1)	8 (19.5)	0.42*	0.310
Dyslipidaemia	55 (51.9)	12 (29.3)	6.10*	**0.036**
Obesity (BMI >30)	54 (50.9)	20 (48.8)	0.06*	0.396
Other comorbidities	64 (60.4)	29 (70.7)	1.36*	0.230

Significant *P* values in bold.

* Independent samples *t* test.

† Mann-Whitney *U* test.

‡ Chi-square test.

## DISCUSSION

Our findings, albeit with a modest sample size, revealed that while diabetes, obesity, hypertension, and dyslipidemia were prevalent among people with TRS, there was no significant difference in the prevalence of metabolic disorders, except dyslipidemia, among those being treated with clozapine or other antipsychotics. Similarly, we did not find a significant difference in metabolic parameters such as fasting blood sugar, weight, BMI, blood pressure, and lipids among those people with TRS who were treated with clozapine or other antipsychotics. Several previous reviews have concluded that clozapine has one of the worst metabolic profiles among antipsychotics and causes widespread metabolic derangements. It is possible that differences in methodology, wherein we compared metabolic problems associated with clozapine and other antipsychotics among a similar cohort of patients (those with TRS) rather than comparing antipsychotics given for varying severity and nature of schizophrenia, could partially account for the discrepant findings. While the clinical indication for clozapine is for people with TRS, other antipsychotics are often used for a broader group of patients with schizophrenia or psychosis of varying severity, including many who have a more favorable response to treatment. Recent research evidence suggests that TRS is a categorically distinct form of schizophrenia or lies at the more severe end of a spectrum of severity among schizophrenia disorders.^26,29^ It could be argued that the more severe psychosocial dysfunctions, substance abuse, severity of illness, cognitive deficits, dysfunction of community functioning, and presence of inflammatory markers associated with TRS compared with treatment-responsive schizophrenia^24–26^ could increase the risk of metabolic problems among people with TRS, irrespective of the nature of antipsychotics used for treating this condition. While studies comparing the diet and exercise of people with TRS and non-TRS are lacking, it is possible that due to the greater severity of the illness, psychosocial adversities, comorbidities, and cognitive problems, compared with people with treatment-responsive schizophrenia, those with TRS might have a more impoverished lifestyle including unhealthy diet, lack of exercise, substance abuse, and exposure to chronic stress, all of which are risk factors for metabolic problems.^4–7^ Furthermore, whether overlapping genetic and inflammatory risk factors between metabolic syndrome, diabetes, obesity, and schizophrenia are more pronounced among people with TRS rather than treatment-responsive schizophrenia has not been adequately investigated.^8,9^

The high rates of dyslipidemia (45.6%), obesity (37.4%), diabetes (22.1%), and hypertension (16.3%) found in our cohort of TRS patients, irrespective of the nature of antipsychotics used, point toward the increased vulnerability of people with TRS to metabolic problems. An umbrella review of 5 meta-analyses of the prevalence of type 2 diabetes among people with schizophrenia found a rate of 10.05%, which is less than half the prevalence of diabetes in our sample.^30^ Similarly, another meta-analysis of 120 studies, the majority from high-income countries, found that the pooled prevalence of obesity in people with SMI was 25.9%, significantly lower than 37.4% in our study.^31^ To our knowledge, studies that focus on metabolic problems among people with TRS receiving treatment with various antipsychotics are lacking. Antipsychotic polypharmacy was common in our cohort of patients with TRS, with 80.5% of the non-clozapine cohort and 55.7% of the clozapine cohort being treated with more than 1 antipsychotic concurrently. While it could be argued that the higher rate of antipsychotic polypharmacy in the non-clozapine group enhanced the metabolic problems among those patients, there is conflicting evidence on the association between antipsychotic polypharmacy and metabolic syndrome in schizophrenia, and no firm conclusion can be made either way on the relationship between antipsychotic polypharmacy and metabolic problems among people with TRS.^32^

Our finding that people with TRS treated with clozapine do not exhibit more significant metabolic problems is supported by a few other studies. A study from Singapore of 88 non-clozapine antipsychotic users and 166 clozapine users found that those in the non-clozapine group had significantly higher diastolic blood pressure, waist circumference, LDL-cholesterol, fasting glucose, and total cholesterol readings.^33^ They also found that the prevalence rates of metabolic syndrome in the clozapine and non-clozapine antipsychotic groups were not statistically significant at 42.8% and 43.2%, respectively. Similarly, another study, which compared 119 patients treated with clozapine and 116 patients treated with long-acting injection antipsychotic medications, did not find significant differences in HbA1C, lipids, and metabolic syndrome between the groups.^34^ However, in both these studies, the proportion of people with TRS among the participants who were not managed on clozapine is unclear. Further, multicentric prospective studies directly comparing the metabolic problems among people with TRS treated with clozapine and other antipsychotics would provide greater clarity on any unique and differential association of clozapine with metabolic issues among people with TRS.

There are several clinical implications for our findings that metabolic problems were prevalent among patients with TRS, with no significant difference in the rates between the clozapine and non-clozapine groups, except for dyslipidemia. In everyday clinical practice, there is extensive evidence that the general medical care of patients with severe mental illness is suboptimal and often neglected.^35^ Due to the nature of their psychotic, negative, cognitive, and other symptoms, unhealthy lifestyle, functional impairments, and lack of funding and policy directives, people with TRS might not be able to access timely and appropriate care for their metabolic problems. The significant proportion of patients with metabolic problems who were being treated with antihypertensives, statins, and antidiabetics among our cohort could be partially explained by the regular medical reviews at the Clozapine Clinic by psychiatrists, close liaison with general practitioners, and 6-monthly metabolic monitoring of the patients. Regular reviews, active monitoring, psychoeducation, and rapport building would assist with the early detection and optimal management of metabolic problems among people with TRS.

While clozapine is the antipsychotic universally recommended by treatment guidelines of schizophrenia for managing TRS and has demonstrated superiority over other antipsychotics for treating TRS,^21,36^ it is under-prescribed in many parts of the world,^37^ and its initiation is often delayed by many years.^38^ Concern among patients and clinicians that clozapine causes worse metabolic side effects than most other antipsychotics could be contributing to its underutilization for people with TRS, along with other factors.^39,40^ If our findings of lack of significant difference in many metabolic problems among patients treated with clozapine and non-clozapine antipsychotics are replicated through more extensive studies, it should allay the fear that clozapine is the main offender in the genesis of metabolic problems among people with TRS, and empower clinicians and patients to take a balanced view about the prescription of clozapine. Furthermore, there is robust evidence from previous research that people with schizophrenia treated with clozapine have greater adherence to the medications used for the treatment of metabolic problems and have a longer life expectancy than those who were not treated with antipsychotics or treated with other antipsychotics.^3,41^ In addition, the findings from our study and previous research of better clinical outcomes among people with schizophrenia treated with clozapine, such as reduced frequency of self-harm, aggression, substance abuse, and hospitalization, compared with those treated with other antipsychotics, are reassuring.^42–45^ A greater focus on addressing unhealthy lifestyles and treating metabolic problems optimally among people with TRS would be appropriate rather than delaying the commencement of clozapine based on concern about metabolic adverse effects. Several dietary, exercise, behavioral, and pharmacological interventions have been trialed with partial success in the management of metabolic disorders among people with schizophrenia.^46–49^

Our findings demonstrated a significantly increased risk of dyslipidemia among people with TRS managed on clozapine. This calls for regular monitoring of lipids and cardiovascular health and appropriate interventions for people treated with clozapine. While 52% of the patients treated with clozapine were diagnosed with dyslipidemia, it is reassuring to note that 75% of these patients were receiving statin treatment. The mean cholesterol level in the clozapine group of 4.8 mmol/L was below the cut-off value for hypercholesterolemia (>5.4) and only 0.4 mmol/L more than in the non-clozapine group. The use of statins in ¾ of patients with dyslipidemia in the clozapine group could possibly account for the normal mean value of cholesterol among the patients who were managed on clozapine. The monthly monitoring of the patients, regular liaison with general practitioners, and 6-monthly monitoring of blood glucose and lipids would have facilitated early detection and treatment of metabolic problems. Dyslipidemia can lead to cardiovascular and cerebrovascular complications and excess deaths among patients with schizophrenia.^3,4^ However, some recent reviewers have pointed out a complex relationship between cholesterol and psychosis, with an association between elevation of cholesterol and improved symptoms of psychosis and increased risk of violence, self-harm, and suicide among people with psychosis with low cholesterol and other lipid levels.^50,51^

### Strengths and Limitations

This study has notable strengths, including being one of the first studies comparing metabolic problems of people on treatment with clozapine and non-clozapine antipsychotics in a similar cohort of chronic patients with TRS. A senior psychiatry trainee registrar meticulously compiled the data under a psychiatrist’s oversight. However, the study has certain limitations. The study, while from a large metropolitan health service, is from a single site, and the sample size is modest, raising the probability of making a Type II error. However, a post-hoc power calculation shows that the current sample size is sufficient (power >80%) to detect a medium effect size. While the participants in the non-clozapine group had ceased clozapine for a long duration (average duration of 5.5 y) and a minimum of 6 months, they had some exposure to clozapine previously. It could be argued that metabolic abnormalities that manifested while on treatment with clozapine persisted in the non-clozapine group, even after a lengthy period of discontinuation. Including a clozapine-naïve group of patients with TRS would have added value to the study. The metabolic and treatment-related data were collected cross-sectionally, so the impact of treatments and other variables over time longitudinally could not be ascertained. We did not collect measurements of weight, blood sugar, lipids, blood pressure, and metabolic disorders before the commencement of clozapine and non-clozapine antipsychotics in the TRS groups. Such data would have added further context to the findings. Our study sample was drawn from a chronic cohort of patients, and the findings cannot be generalized to patients with first-episode schizophrenia with TRS who are managed on clozapine and other antipsychotics.

## CONCLUSIONS

The findings of our study suggest that among chronic patients with TRS, metabolic disorders such as obesity, diabetes, and dyslipidemia are prevalent. A comprehensive approach addressing a broad range of risk factors and their management, rather than solely focusing on clozapine as the source of the metabolic problems, would be appropriate. Hesitation to commence clozapine merely based on concern about metabolic problems is not warranted in many instances. Other risk factors for metabolic disorders, such as unhealthy diet, lack of exercise, genetic overlap, and the presence of inflammatory processes, warrant greater scrutiny in clinical practice and research among people with TRS.
